# Workplace violence (WPV) in healthcare systems

**DOI:** 10.1002/nop2.713

**Published:** 2020-11-27

**Authors:** Mahboubeh Dadfar, David Lester

**Affiliations:** ^1^ School of Behavioral Sciences and Mental Health‐Tehran Institute of Psychiatry, International Campus, School of Public Health Student Committee of Education and Development Center (EDC) Iran University of Medical Sciences Tehran Iran; ^2^ Stockton University School of Social and Behavioral Sciences Galloway NJ USA

## 1 WORKPLACE VIOLENCE (WPV) IN HEALTHCARE SYSTEMS

Workplace violence (WPV) is the third major cause of death due to occupational injuries in the United States and the second major cause of female mortality in the workplace. WPV refers to assault and other violent acts or threats associated with the workplace, resulting in physical and emotional harm to the individual and damage to resources (Boafo, 2018). WPV is very common in healthcare systems and can result in mental health problems for the victims. It leads to anxiety, post‐traumatic stress disorder (PTSD), distrust, loss of self‐confidence, low self‐esteem, increased job stress, absence from work, job dissatisfaction, leaving the workplace, occupational and mental burnout, problems in interpersonal relationships, an increase in clinical and therapeutic errors, disability and suicide. In healthcare systems, WPV also has an impact on the patients' security and safety (Havaei et al., 2019).

Although violence occurs in all workplaces, nurses, with their close contact with the patient and their relatives, are three times more likely to be at risk of violence than other healthcare personnel. WPV can change the nurses’ attitude towards the profession of nursing and reduce their motivation, quality of care and career satisfaction (Boafo, 2018; Duan et al., 2019; Li et al., 2020; Liu et al., 2018; Tian et al., 2020).

The incidence of physical WPV ranged from 4.9%–83.3% and verbal WPV from 66.2%–95.1% in the prior year among nurses (Jakobsson et al., 2020; Shi et al., 2020). Regards to association between gender and physical and verbal WPV, prior findings found are contradictory (Acquadro Maran et al., 2019; Li et al., 2020).

For various reasons, including lack of support from hospital management, fear of revenge, lack of knowledge of nurses about WPV and ignorance of legal strategies to deal with WPV, the rate of WPV is probably underreported (Hedayati Emam et al., 2018). Trends in the prevalence of this problem over time are unknown in Iran (Esmaeilpour et al., 2011; Rezaei nayeh et al., 2018). The main reasons for dissatisfaction with how WPV was handled were related to nurse‐authority disagreement, lack of personnel, lack of medications, rules and regulations, lack of security and no violence management training for WPV.

Overall, results indicate a need to develop a more positive work culture by holding WPV management teams and establishing appropriate regulations for improving workplace safety for nurses, which will also improve the quality of patient care. Education in the prevention and control of violence and evidence‐based interventions will reduce the risk of victimization by violence. WPV against nurses should also be studied from the patients’ perspective. WPV by staff against one another should also be investigated.

It is concluded that policies and laws must be implemented. Providing security systems and the formulation of policies and procedures for the prevention of violence are necessary for the WPV. The policies and strategies should be sensitive to local conditions and needs. Reporting WPV and counselling for nurses who are victimized are needed to encourage the reporting of WPV and providing medical care after a victimization.

## CONFLICT OF INTEREST

Authors declare that they have no conflict of interest.

## AUTHOR CONTRIBUTIONS

All authors have agreed on the final version and meet at least one of the following criteria [recommended by the ICMJE (http://www.icmje.org/recommendations/)]: substantial contributions to conception and design, acquisition of data or analysis and interpretation of data; and drafting the article or revising it critically for important intellectual content.

## Data Availability

The data sets generated during and/or analysed during the current study are available from the corresponding author on reasonable request.
